# Unoccupied Buildings and Vacant Beds

**Published:** 1911-10-28

**Authors:** 


### HOSPITAL ECONOMICS.

### Unoccupied Buildings and Vacant Beds.

There is nothing less satisfactory than to have buildings erected at great cost for hospital purposes, as in the case of St. Mary's Hospital and in some of the Metropolitan Asylums Board and other public hospital buildings, except perhaps the scandal of so overcrowding the beds of a given hospital as to make it possible that it may become necessary to refuse an urgent case of illness or accident for lack of a bed to place the patient in. So far as voluntary hospitals are concerned, where buildings have been erected and then left unoccupied for lengthened periods, the fault has lain at the feet of those who in their enthusiasm for extensions have persisted in going on with such buildings before the means for their support was forthcoming, or any practical scheme whereby it could be obtained was in active working. Not only is there the scandal and unpopularity of having fine buildings unoccupied when attached to a hospital, but the waste of such ill-judged expenditure on bricks and mortar is continuously increasing from the circumstance that unoccupied buildings have a way of rapidly degenerating, structurally asd otherwise. Fortunately the institution of the King's Hospital Fund has largely diminished the risk of improvident buildings for the future, so far as the Metropolitan Voluntary Hospitals are concerned.

In the case of the Metropolitan Asylums Board Hospitals which have been maintained ready for use but unoccupied for lengthened periods of time, there has been the justification that at the time they were erected public opinion required such provision owing to the absence of the present high efficiency and fuller knowledge in regard to the power of preventive measures to diminish the risks of epidemics. It is a remarkable fact, and one which is as honourable to the man as it is pleasant to the journalist to record, that one member of the present Ministry, Mr. John Burns, has steadily and energetically worked to master the varied and difficult ramifications of the organisations and questions which appertain to his department, until he has obtained a grip of the whole facts and their meaning, which few if any ministers have ever attained to in this generation. The results have been of grave importance to the welfare of all classes of the community, and to the economical administration of hospitals and many buildings erected for public purposes, which have come under his jurisdiction. He has evolved the wise policy that before more money is spent upon bricks and mortar, the existing buildings of every kind which may be unoccupied shall so far as practicable be utilised everywhere to avoid capital outlay and secure economical administration. To quote one line of policy out of several as an example of what we here state, we may refer to the action of the Local Government Board in furthering the opening of the Metropolitan Asylums Board Hospital at Beddington, and the taking over of the Fever Hospital buildings at Hither Green, so as to convert both establishments into great institutions for the reception of poor children where they can be treated and educated apart from all the surroundings which make for less than the best, in regard to character and a standard of personal conduct in the young. It has been well said that almost everything that is great has been done by youth. These children under a proper system, and the influences which will surround them in the great institutions referred to, should when the time comes be as well equipped as possible to face the world and make their living. They may be numbered among the most useful citizens of the community.

There has been during recent years almost a craze for the erection of new buildings. Under the Insurance Bill, as it stands, a very large sum amounting to hundreds of thousands of pounds is provided for the erection of sanatoria all over the country. We hope, and judging by his record we believe, that in this we shall have the support of the President of the Local Government Board, that before any sanatoria buildings are erected care must be taken in every district to strictly classify the class of cases which it is proposed to deal with in sanatoria. Next the right policy will be first of all to utilise, by necessary conversion, all the unoccupied buildings which the State or local authorities have available, and then to determine how, and where, and when, new sanatoria shall be built. If this be done the credit which is attached to the administration of Mr. John Burns in regard to policy will be shared by the Chancellor of the Exchequer, and both may be proud that they have been given the wisdom to make it their business, as a first duty, to protect the interests of the State while providing the fullest facilities for the comfort, and protection of the citizens in the day of disease.

Unoccupied beds must be considered from three points of view. First, every well-administered voluntary hospital must exhibit sufficient strength, through its administrators, to decline to attempt to do more work than their accommodation enables them to undertake and to do, with the maximum of efficiency. Every hospital which is wisely administered invariably has a few beds always vacant for cases of pressing emergency brought in at any time during the twenty-four hours. In such beds urgent accidents and cases of a doubtful character can be retained until their exact requirements in regard to hospital care are ascertained and made clear.

Secondly, no one with a knowledge of the working of a well-administered hospital will regard any bed left unoccupied as a vacant bed, in the sense that it is desirable to fill it at once with a patient, providing the actual number of such vacant beds are sufficient merely, having regard to the character and size of the hospital, to provide that no really urgent case need ever be refused admission. Fortunately this simple rule has now come to be generally observed, and hospital scandals in consequence have become as rare as they are easily preventable.

Thirdly, where a hospital or other institutional building is proved by the claims which are made upon it by patients to be in excess of such requirements, this vacant accommodation should be scheduled, and care should be taken to utilise it to the best advantage for objects which come within the scope of Government departments like that of the Local Government Board. In the case of the hospitals in a great city like London, the King's Fund has shown how large a sum can be saved in construction, by first securing that all beds really vacant are filled, before further extension is sanctioned by those who have to provide a good proportion or the whole of the money involved. It will thus be seen that the economical side of bricks and mortar here dealt with results, under a wise administration, in the saving of large sums of public and charitable funds, to the increased reputation of Governments and administrators, and the general efficiency of the establishments where such economy has been seriously enforced.

